# Effects of Persistent Atrial Fibrillation-Induced Electrical Remodeling on Atrial Electro-Mechanics – Insights from a 3D Model of the Human Atria

**DOI:** 10.1371/journal.pone.0142397

**Published:** 2015-11-25

**Authors:** Ismail Adeniran, David H. MacIver, Clifford J. Garratt, Jianqiao Ye, Jules C. Hancox, Henggui Zhang

**Affiliations:** 1 Biological Physics Group, School of Physics and Astronomy, University of Manchester, Manchester, United Kingdom; 2 Taunton & Somerset Hospital, Somerset, United Kingdom; 3 Manchester Heart Centre, Manchester Royal Infirmary, Manchester, United Kingdom; 4 Department of Engineering, Lancaster University, Lancaster, United Kingdom; 5 School of Physiology and Pharmacology, and Cardiovascular Research Laboratories, University of Bristol, Bristol, United Kingdom; Gent University, BELGIUM

## Abstract

**Aims:**

Atrial stunning, a loss of atrial mechanical contraction, can occur following a successful cardioversion. It is hypothesized that persistent atrial fibrillation-induced electrical remodeling (AFER) on atrial electrophysiology may be responsible for such impaired atrial mechanics. This simulation study aimed to investigate the effects of AFER on atrial electro-mechanics.

**Methods and Results:**

A 3D electromechanical model of the human atria was developed to investigate the effects of AFER on atrial electro-mechanics. Simulations were carried out in 3 conditions for 4 states: (i) the control condition, representing the normal tissue (state 1) and the tissue 2–3 months after cardioversion (state 2) when the atrial tissue recovers its electrophysiological properties after completion of reverse electrophysiological remodelling; (ii) AFER-SR condition for AF-remodeled tissue with normal sinus rhythm (SR) (state 3); and (iii) AFER-AF condition for AF-remodeled tissue with re-entrant excitation waves (state 4). Our results indicate that at the cellular level, AFER (states 3 & 4) abbreviated action potentials and reduced the Ca^2+^ content in the sarcoplasmic reticulum, resulting in a reduced amplitude of the intracellular Ca^2+^ transient leading to decreased cell active force and cell shortening as compared to the control condition (states 1 & 2). Consequently at the whole organ level, atrial contraction in AFER-SR condition (state 3) was dramatically reduced. In the AFER-AF condition (state 4) atrial contraction was almost abolished.

**Conclusions:**

This study provides novel insights into understanding atrial electro-mechanics illustrating that AFER impairs atrial contraction due to reduced intracellular Ca^2+^ transients.

## Introduction

Atrial fibrillation (AF) is the most common sustained cardiac arrhythmia [1,2] and has an increase in incidence and prevalence with each decade of adult life [3,4]. AF may be precipitated by a variety of cardiac or non-cardiac diseases which cause abnormalities in cardiac electrophysiology and in turn act as a substrate for the development of the arrhythmia [5]. Current treatments for atrial fibrillation to restore sinus rhythm include external and internal Direct Current (DC) cardioversion, chemical cardioversion (pharmacological intervention), and radiofrequency ablation [1–4].

Atrial stunning is the loss of mechanical atrial contraction following a successful cardioversion, which is maximal immediately after cardioversion and can take up to 6 weeks for normal atrial contraction to re-establish [6]. A long period of atrial stunning may cause an increased risk of thromboembolism.

Atrial stunning occurs rarely following spontaneous cardioversion in paroxysmal arrhythmias. Previous studies have also shown that factors delaying return of normal atrial mechanical function include duration of atrial fibrillation, presence of structural heart disease, atrial pressures and atrial size [6–8]. However, the exact mechanisms causing impaired atrial mechanics, as occurs in atrial stunning are unknown. Some postulated mechanisms include tachycardia induced atrial cardiomyopathy, accumulation of cytosolic calcium and atrial hibernation [6–8].

It is unknown why atrial stunning occurs frequently for chronic AF but rarely for paroxysmal AF patients [6–8]. The intrinsic electrophysiological properties of the atria are altered during chronic AF due to the atrial fibrillation induced electrical remodelling (AFER) [9–13]. Multiple clinical electrophysiological and experimental studies have shown that electrical remodelling is characterised by an abbreviated atrial action potential (AP) morphology, which is associated with underlying changes to the density and kinetics of some membrane ionic currents and to cellular Ca^2+^ handling processes [10–12,14–16]. Chronic atrial fibrillation can also cause atrial structural remodelling, which is characterised by down-regulation and heterogeneous expression of connexin proteins that form intercellular gap junctions (responsible for the AP conduction), as well as the presence of severe fibrosis, accumulation of fatty deposits and fibre disorganisation [9,10,13,17–19]. All of these factors may contribute to decreases in the AP conduction velocity and increases in conduction anisotropy and heterogeneity.

We hypothesised that the impaired atrial mechanics as seen in atrial stunning after a successful cardioversion to chronic atrial fibrillation might be due to AFER during chronic fibrillation, which impairs the mechanical contraction of the human atria. However, due to the complexity of the atrial system, the functional impact of AFER on atrial electro-mechanical dynamics has not yet been investigated in such a way that the ionic mechanisms underlying atrial stunning can be elucidated. Though previous electrophysiological studies have identified some cellular and sub-cellular changes associated with chronic AF, such as abbreviated action potential duration (APD) and reduced amplitude of the intracellular Ca^2+^ transient [10–12,14–16], changes at the whole organ level emerge from both cellular (i.e, single cell behaviours) and intercellular (i.e., cell-to-cell interactions) dynamic processes.

Computational models provide a powerful tool to study cardiac function [20–23]. Being constructed from and validated against experimental data, they provide a means for quantitatively predicting the functional roles of altered molecular dynamics and ionic channels on cardiac functions in a systematic fashion that is difficult to achieve in an experimental setting. Therefore, in this study, we developed a novel 3D anatomical model of the human atria with coupled electrical and mechanical dynamics at cellular and tissue levels. Using the multi-scale models we investigated the functional impact of AFER on atrial electrical and mechanical activities in order to elucidate the mechanism underlying atrial stunning. The principal contributions of this study are: (i) the development of a new family of biophysically detailed, electrical-mechanically coupled models of the human atrium at cellular and 3D anatomical levels; (ii) investigation of the functional impacts of AFER on the electrical and mechanical dynamics of the atria; and (iii) elucidation of possible mechanisms underlying the impaired mechanical contraction as seen in atrial stunning in patients with persistent atrial fibrillation.

## Materials and Methods

### Regional Single Cell Models

**Fig 1:**
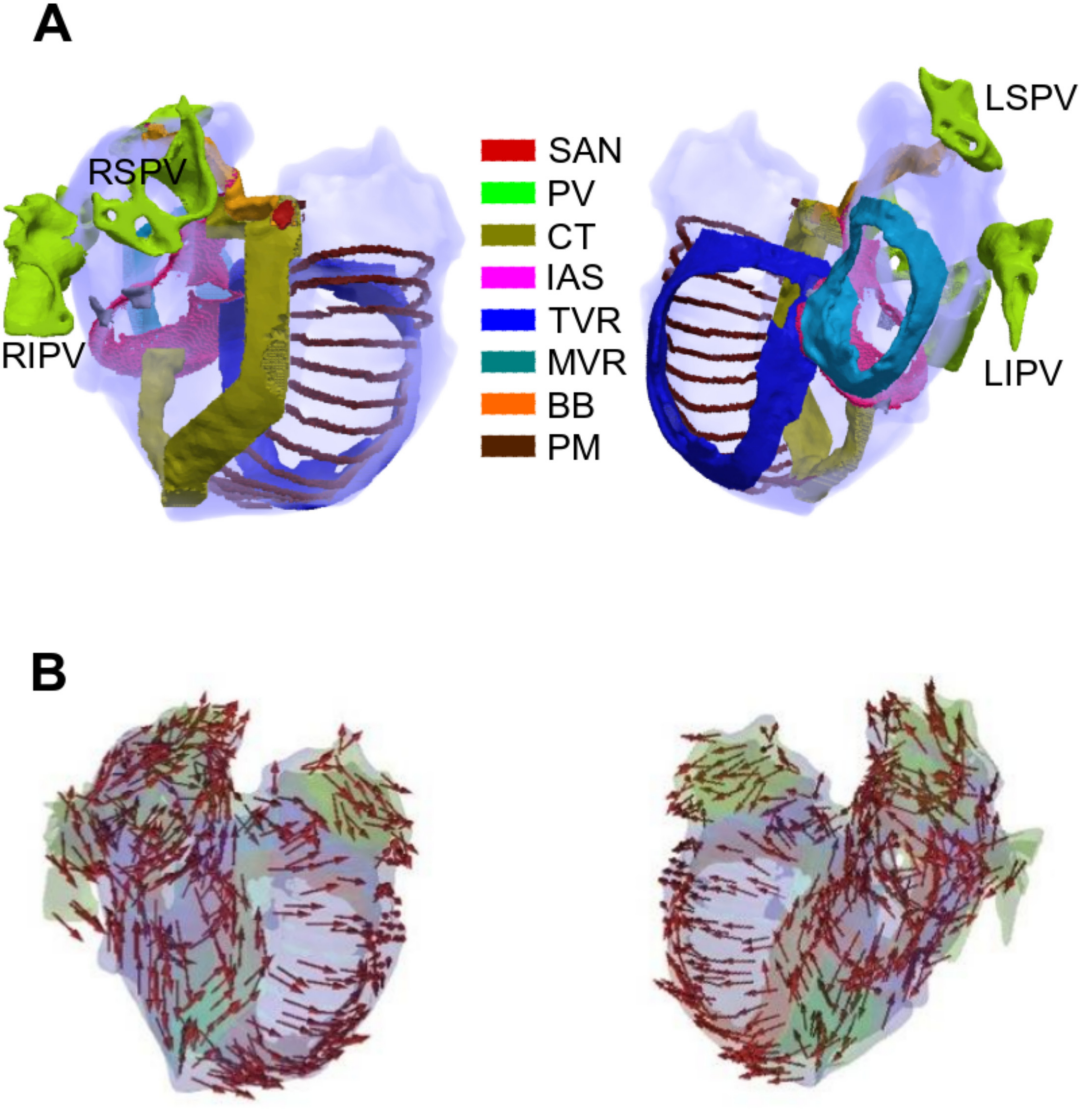
3D anatomical atrial geometry. (A) Segmented atria from two different views (left: top view and right: view into the atrial cavities). All regions are labelled. (B) Fibre orientations of the atria from two different views (left: top view and right: view into the atrial cavities). SAN: Sinoatrial Node, PV: Pulmonary Vein, CT: Crista Terminalis, IAS: Inter-atrial Septum, TVR: Tricuspid Valve Ring, MVR: Mitral Valve Ring, BB: Bachmann’s Bundle, PM: Pectinate Muscle, RS (Right Superior), RI (Right Inferior), LS (Left Superior), LI (Left Inferior).

The atria are composed of several electrically distinct regions (Fig 1A). This regional electrical heterogeneity is thought to play a large role in the genesis and maintenance of atrial arrhythmias [24–26]. Data for human atrial electrophysiology are scarce, particularly for action potential variations in the different regions within the atria. Most studies involving the investigation of the ionic mechanisms underlying regional action potential variations in the atria have been carried out on other mammals, mainly dog [27–30].

In this study, we modified the well established Courtemanche-Ramirez-Nattel (CRN) model [31] of the human atrial cell to couple electrophysiology to mechanics. As the CRN model provides detailed descriptions of cellular electrical APs in the right atrium (RA), the model was first modified to incorporate experimental data from the literature [27–29] on the heterogeneous ionic currents across the atria as implemented in previous modelling studies [32,33]. Relative changes in maximum ion current conductances for the different regions of the atria are listed in Table 1.

**Table 1:** Scale factors ion channel conductivities for the different regions of the atria relative to the base model of [31]. Modified from [32].

Heterogeneity	Source	G_Na_	G_to_	G_CaL_	G_Kr_	G_Kur_
**RA/PM**	Base model	1.00	1.00	1.00	1.00	1.00
**CT upper endo**	[29]	1.00	1.00	1.67	1.00	1.00
**CT upper epi**	[27]	1.00	0.50	1.67	1.00	1.00
**CT lower endo**	[27]	1.00	0.68	1.67	1.00	1.00
**CT lower epi**	[27]	1.00	0.34	1.67	1.00	1.00
**BB (RA part)**	[27,29]	1.00	1.00	1.67	1.00	1.00
**TVR**	[29]	1.00	1.00	0.67	1.53	1.00
**MVR**	[29,34]	1.00	1.00	0.67	2.44	1.00
**RAA**	[29]	1.00	0.68	1.06	1.00	1.00
**LAA**	[29,34]	1.00	0.68	1.06	1.60	1.00
**LA**	[34]	1.00	1.00	1.00	1.60	1.00
**SEP**	[35]	1.50	1.00	0.25	1.00	0.67

RA: Right Atrium PM: Pectinate Muscle, CT: Crista Terminalis, endo: endocardium, epi: epicardium, BB: Bachmann’s Bundle, TVR:Tricuspid Valve Ring

Specifically, the conductances of the following ion channel currents were rescaled based on experimental data [27,29,34,35] to reproduce AP morphologies in various parts of the atria: L-type Ca^2+^ current (I_CaL_), transient outward K^+^ current (I_to_), rapidly-activated potassium current (I_Kr_), ultra rapid delayed rectifier current (I_Kur_) and the fast Na^+^ current (I_Na_). The scale factors of the conductances in the RA and left atrium (LA), Bachmann’s bundle (BB), crista terminalis (CT), pectinate muscles (PM), right and left atrial appendages (RAA and LAA respectively), tricuspid and mitral valve rings (TVR and MVR respectively) and atrial septum are summarised in Table 1.

Then the CRN model was modified to incorporate the electrical-mechanical coupling. Due to the lack of detailed experimental data on which to base the development of biophysically detailed equations for the electrical-mechanical coupling of the human atrial myocytes, this was done by coupling the CRN electrophysiological model to the Rice *et al*. myocyte contraction model [36], which describes the mechanics of a cardiac myocyte. This model was chosen as it is based on the cross-bridge cycling model of cardiac muscle contraction and is able to replicate a wide range of experimental data including steady-state force-sarcomere length (F-SL), force-calcium and sarcomere length-calcium relations [36]. The Rice *et al*. myocyte contraction model [36] was developed for guinea-pig myocytes. In order to validate its use for human atrial myocytes, we followed our previous studies [20,37–39] to simulate the force-calcium relationship in the modified CRN model with incorporation of the Rice *et al*. electrical-mechanical coupling equations. The simulated data replicated the experimental data on the force-calcium relationship from human atrial myocytes [40].

As in our previous studies [20,37–39], the intracellular calcium concentration [*Ca* ^2+^]_*i*_ from the single cell electrophysiology models (EP) was used as the coupling link to the myofilament mechanics model (MM). [*Ca* ^2+^]_*i*_ produced as dynamic output from the EP models during the AP served as input to the MM model from which the amount of calcium bound to troponin is calculated. The resulting formulation of the myoplasmic Ca^2+^ concentration in the electromechanically coupled model is:

$$\frac{dCa_{i}}{dt}=\frac{\frac{-C_{m}\cdot \left(I_{CaL}+I_{bCa}+I_{pCa}-2I_{NaCa}\right)}{2V_{myo}F}+\frac{-V_{nsr}\cdot \left(I_{leak}-I_{up}\right)+0.5I_{rel}V_{jsr}}{V_{myo}}-\frac{dTroptot_{Ca}}{dt}\times \frac{1}{1000}}{\frac{1+{\left[CMDN\right]}_{max}K_{m,CMDN}}{{\left({\left[Ca^{2+}\right]}_{i}+K_{m,CMDN}\right)}^{2}}}$$ (1)

where *C*_*m*_ is the membrane cell capacitance per unit surface area, *V*_*nsr*_ is the network sarcoplasmic reticulum (SR) volume, *V*_*jsr*_ is the junctional SR volume, *V*_*myo*_ is the cytoplasmic volume, *I*_*leak*_ is the SR Ca^2+^ leak current, *I*_*up*_ is the Ca^2+^ uptake current into the NSR, *I*_*bCa*_ is the background Ca^2+^ current, *I*_*pCa*_ is the sarcoplasmic Ca^2+^ pump current, *I*_*NaCa*_ is the Na^+^/Ca^2+^ exchanger current, *I*_*CaL*_ is the L-type inward Ca^2+^ current, *I*_*rel*_ is the Ca^2+^ release current from the JSR, *F* is the Faraday constant, *[CMDN]*_*max*_ is the total calmodulin concentration in the myoplasm, *K*_*m*,*CMDN*_ is the Ca^2+^ half-saturation constant for calmodulin and $\frac{dTropto_{Ca}}{dt}$ is the rate of Ca^2+^ binding to troponin.

To model the effect of electrophysiological remodelling associated with AF, we followed the work of Colman *et al*. [41], which was based on an extensive review of the available experimental data regarding AF remodelling of the main ion channels and sarcoplasmic reticulum processes. Table 2 summarises the modifications made to the control/healthy electromechanical single cell models to generate a family of AF-remodelled single cell models.

**Table 2:** Implementation of AF remodelling.

Process	Model
I_CaL_	-70%
I_Kur_	-50%
I_to_	-65%
I_K1_	+100%
I_Ks_	+100%
I_KAch_	No change
I_NaCa_	+55%
I_Kr_	No change
SERCA	+50%
RyR	+300%
SR Ca^2+^ leak	+25%

Maximum channel conductances of the control model were changed based on an extensive review of available experimental data following the work of [41]. SERCA = Sarco/endoplasmic Ca^2+^-ATPase.

### 3D tissue model of electro-mechanical coupling

3D anatomical model: The 3D anatomical model was reconstructed and segmented based on anatomical features from the Visible Female dataset [42] (Fig 1A). As the intrinsic heterogeneity of electrophysiological properties of atrial tissue plays an important role in ensuring the right timing sequences of depolarization and repolarization pattern in the atria, the 3D model considers different electrical properties for different regions of the atria: the sinoatrial node (SAN), left atrium (LA), right atrium (RA), crista terminalis (CT), pectinate muscles (PM), limbus fossa ovalis, Bachmann’s bundle (BB), right inferior isthmus, pulmonary veins (PV), right atrial appendage (RAA), left atrial appendage (LAA), inter-atrial septum (SEP), tricuspid valve ring (TVR) and mitral valve ring (MVR). Fibre orientation was incorporated using a novel semi-automatic rule-based approach and was validated against patient-specific volumetric models derived from CT, MRI and photographic data [43,44] (Fig 1B).

3D electrophysiological model: Electrical excitation wave propagation in the 3D human atria was modeled by a monodomain representation [20,45–47] of cardiac tissue with a modification (i.e., the incorporation of the Right Cauchy Green deformation tensor, ***C***) to take into account the effect of the deforming atrial tissue, similar to previous studies [20,37,48–50].

$$C_{m}\frac{dV}{dt}=-\left(I_{ion}+I_{stim}\right)+\nabla \cdot \left(DC^{-1}\nabla V\right)$$ (2)

where *C*_*m*_ is the cell capacitance per unit surface area, *V* is the membrane potential, *I*_*ion*_ is the sum of all transmembrane ionic currents from the electromechanics single cell model, *I*_*stim*_ is an externally applied stimulus and *D* is the diffusion tensor. The tissue conductivities that make up the diffusion tensor, *D*, were chosen so that the activation time and conduction velocities of the atria matched those of well established atrial models [26,32,51]. The conductivity ratio (parallel to fibre direction:transverse direction) was set to 3:1. The longitudinal (along the fibres) and transverse conductivities were 1.26 mm^2^ ms^-1^ and 0.42 mm^2^ ms^-1^ respectively reproducing the heterogeneous conduction velocities of well established atrial models [32,51] and human experimental data [52].

3D electro-mechanical coupling model: To develop a 3D electromechanical model of the human atria, the family of electromechanically coupled single cell atrial models developed in this study was incorporated into a 3D atrial geometry.

We modelled cardiac tissue mechanics within the theoretical framework of nonlinear elasticity [53,54] as an inhomogeneous, anisotropic, nearly incompressible material similar to previous studies [49,55–60]. The coupling between cardiac mechanics and electrical excitation propagation is addressed in a framework in which the electrical action potential dictates the active strain of the muscle [58–60]. We adopted an active strain approach in this study as opposed to the active stress approach [48,49,61]. Unlike the active stress approach, the active strain approach requires no tuning to provide the observed deformation when fibre contraction is included in the equations. In addition, frame invariance and rank-one ellipticity are inherited from the corresponding properties of the standard strain energy of the material [58,60,62] whereas rank-one ellipticity cannot be ensured when large deformations occur for a specific active stress form [60,63].

We used a two-field variational principle with the deformation *u* and the hydrostatic pressure *p* as the two fields [54,64,65]. *p* is utilized as the Lagrange multiplier to enforce the near incompressibility constraint. Thus, the total potential energy function Π for the mechanics problem is formulated as:

$$\Pi \left(u,p\right)=\Pi_{\text{int}}\left(u,p\right)+\Pi_{ext}\left(u\right)$$ (3)

where Π_int_(*u*,*p*) is the internal potential energy or total strain energy of the body and Π_int_(*u*) is the external potential energy or potential energy of the external loading of the body.

The deformation gradient *F* is a tensor that maps elements from the undeformed configuration to the deformed configuration [53,54]. Following [58,66], we multiplicatively decompose *F* into a microscopic (active) component and a macroscopic elastic (passive) component:

$$F=F_{e}F_{o}$$ (4)

The active component *F*_*o*_ measures the length change of the tissue due to muscle contraction while the passive component *F*_*e*_ accounts for the passive mechanical response of the tissue and possible tension due to external loads.

With the vector field ***f*** denoting the unique direction of the fibres in the undeformed state of the atria, the microscopic active component of the deformation tensor *F* takes the form:

$$F_{o}=I+\gamma \left(SL\right)f⊗f$$ (5)

where *I* is the identity tensor, *SL* is the sarcomere length of the electromechanical single cell, γ is a scalar field that represents the intensity of the contraction, i.e., the active strain:

$$\gamma =\frac{SL-SL_{0}}{SL_{0}}$$ (6)

where *SL*_*0*_ is the resting sarcomere length. Thus, *γ*>0 denotes elongation, and *γ*<0 denotes contraction.

The elastic component *F*_*e*_ is formulated as:

$$F_{e}=FF_{o}^{-1}$$ (7)

and the corresponding Right Cauchy-Green strain tensor is:

$$C_{e}=F_{e}^{T}F_{e}$$ (8)

The associated Green-Lagrange strain tensor is:

$$E_{e}=\frac{1}{2}\left(C_{e}-I\right)$$ (9)

To characterise the constitutive behaviour of cardiac tissue, for the strain energy function *W*, we used the Guccione constitutive law [67] given by:

$$W=W\left(F_{e}\right)=C_{1}e^{Q}$$ (10)

where:

$$Q=C_{2}E_{11}^{2}+C_{3}\left(E_{22}^{2}+E_{33}^{2}+E_{23}^{2}\right)+2C_{4}\left(E_{12}E_{21}+E_{13}E_{31}\right)$$ (11)

following previous modelling studies [20,68]. *C*_*1*_ = 0.831 kPa, *C*_*2*_ = 14.31, *C*_*3*_ = 4.49 and *C*_*4*_ = 10. *E*_*ij*_ are the components of the Green-Lagrange strain tensor.

We applied a constant pressure boundary condition of 1.07 kPa on the endocardial surface of the atria (Γ_endo_) and to prevent rigid body rotations, we set the displacement ***u*** = *0* on the subset of the epicardial surface (Γ_epi_) in the left atria, which is normally in close proximity to the thoracic spine and between the RIPV and the RSPV (Fig 1).

Solving the 3D electromechanics model: The electromechanics problem consists of two sub-problems: the electrophysiology problem (Eq 2) and the mechanics problem (Eq 3). The electrophysiology problem (Eq 2) was solved with a Strang splitting method [69] ensuring that the solution is second-order accurate. It was discretized in time using the Crank-Nicholson method [70], which is also second-order accurate and discretized in space with Finite Elements [70–73]. *I*_ion_ in (Eq 2) represents the single cell electromechanics model from which the active strain (Eq 6) input to the 3D mechanics model for contraction was obtained. This was done via an L2 projection from the finite element space of the electrophysiology mesh to the finite element space of the mechanics mesh. The system of ordinary differential equations (ODE) composing *I*_ion_ was solved with a combination of the Rush-Larsen scheme [74] and the CVODE solver [75,76].

The mechanics problem (Eq 3) was also solved using the Finite element Method using the automated scientific computing library, FEniCS [77]. The resulting non-linear system of equations was solved iteratively using the Newton-Raphson method to determine the equilibrium configuration of the system. FEniCS [77], which is a problem-solving environment for automated solution of finite element computations allows a close relation between the mathematical notation and the source code, and automated differentiation of both the constitutive law and the residual equation. It provides functionality that can be used to linearize the nonlinear residual equation (linear form) automatically for use with the Newton-Raphson method. Features of the Unified Form Language (UFL) [77,78] used in the FEniCS [77] project permit problems to be posed as energy minimization problems, making it unnecessary to compute an expression for a stress tensor, or its linearization, explicitly. The value of the Right Cauchy Green Tensor *C* was then used to update the diffusion coefficient tensor in Eq 1. Over a typical finite element domain, *P*_2_ elements [71–73] were used to discretize the displacement variable *u*, while the pressure variable *p* was discretized with *P*_1_ elements [71–73]. This *P*_2_–*P*_1_ mixed finite element has been proven to ensure stability [77,79,80] and an optimal convergence rate [73,79,81].

The algorithm for solving the full electro-mechanics problem is as follows:

1. Determine the initial deformation and obtain the value of the Right Cauchy Green Tensor *C*.
2. While time < end time of simulation:
  - Solve the electrophysiology problem for Δt_mechanics_ = 1 ms with *C* as input and active strain γ as output (Δt_electrophysiology_ = 0.01 ms).
  - Project γ from the electrophysiology mesh onto the mechanics mesh.
  - Solve the mechanics problem with γ as input and *C* as output.

Meshes and Computation: The atrial electrophysiology mesh consisted of 2736128 tetrahedra and 566234 vertices while the atrial mechanics mesh consisted of 42752 tetrahedra and 14349 vertices. The two meshes were also checked to ensure good mesh quality metrics. The single cell ODEs were parallelised using OpenMP while the Conjugate Gradient method with the Symmetric successive overrelaxation preconditioner (Electrophysiology) and the parallel sparse direct solver MUMPS (Mechanics) were used for Linear Algebra via PETSc [82,83]. The simulations were carried out on an Intel Xeon CPU E5-2687W @ 3.10 GHz with 32 hyperthreaded cores and 64 GB of memory. It took about 48 hours to simulate a period of 1000 ms electro-mechanical activities of the model.

Methods of stimulus, and measurement of atrial volume: For the single cell simulations, APs were elicited with an S1-S2 protocol consisting of 1000 S1 stimuli followed by a single S2 stimulus at a frequency of 1 Hz. For the 3D simulations, in order to guard against any drift in the steady state values of the ion concentrations in the model, the electromechanical single cell models described in section 2.1 were pre-paced for a 1000 beats before being incorporated into the tissue model.

Atrial volume was computed as a surface integral using the Ostrogradsky formula [84]:

$$V_{INNER}\left(\varphi \right)=\int_{\varphi (\Omega_{INNER})}dv=-\frac{1}{3}\int_{\partial \varphi \left(\Gamma_{ENDO}\right)}x\cdot nds$$ (12)

where φ is the deformation, Ω_*INNER*_ is the volume enclosed by the atrial endocardium Γ_*ENDO*_ and ***n*** is the outward unit normal vector.

### Modelling different states of the atria

In order to investigate the functional impact of AFER on atrial electromechanical dynamics, we implemented 3 simulation conditions mimicking 4 states of atrial tissue. These include: (i) the control condition, which mimics the healthy and normal atrial tissue (state 1), as well as the tissue about 6 weeks after successful cardioversion (state 2). This assumption is reasonable as the atria normally restore their mechanical contraction about 6 weeks after successful cardioversion with atrial stunning [6–8,85]. This time period is sufficient for atrial tissue to go through reverse electrical remodeling after cardiac arrhythmias as shown in some experimental animal studies [86]; (ii) AFER-SR condition that simulates the AF-remodeled atrial tissue with normal sinus rhythm (SR) (state 3). This condition mimics the state of atrial tissue just after a successful cardioversion such that the tissue electrophysiology is remodelled; and (iii) AFER-AF condition simulating the AF-remodeled tissue with multiple reentrant excitation waves (state 4). This condition mimics the atrial tissue during chronic atrial fibrillation, in which both tissue electrophysiological remodeling and re-entrant wavelets are present.

## Results

### Single Cell Electromechanical Simulations

**Fig 2:**
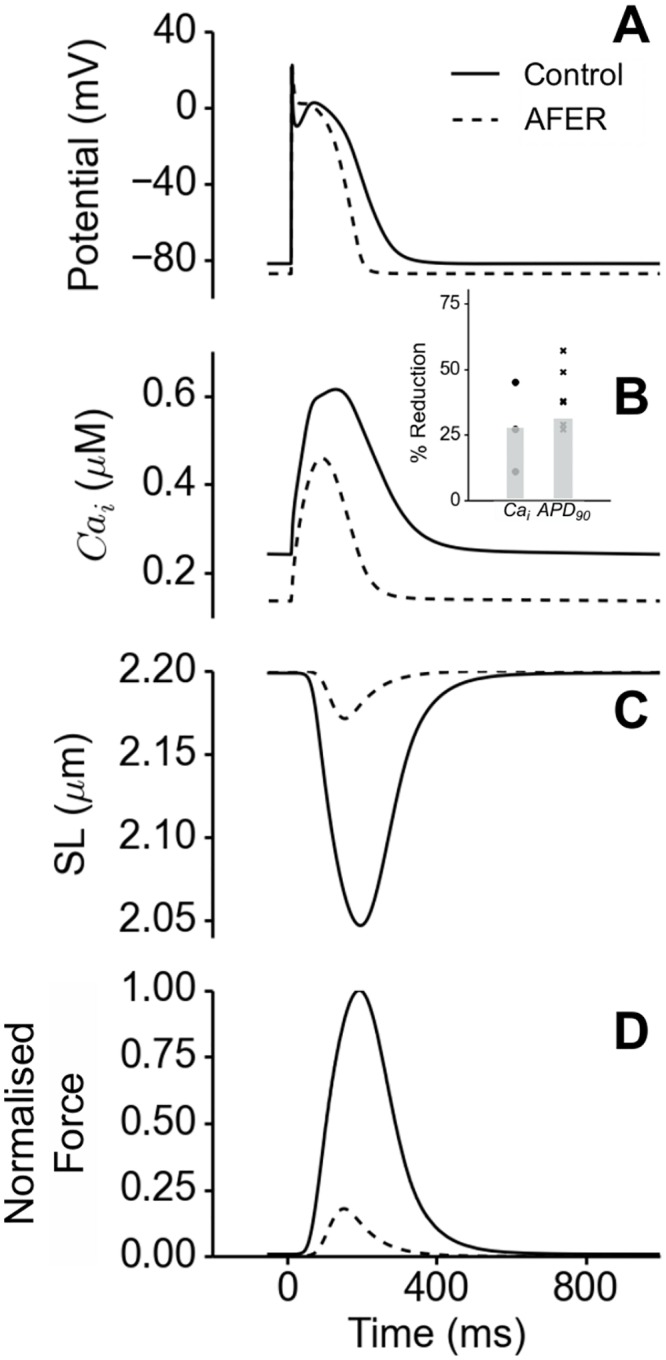
Electromechanical properties of the baseline, isolated RA single cell under control (dark) and AFER (dashed) conditions. (A) Action potential. (B) Cytosolic Ca^2+^ concentration. (**Inset**) Comparison of our simulation results (grey bars) with experimental data (symbols) on APD_90_ [10–12,14,87,88] and cytosolic Ca^2^+ reduction [88,90,91]. (C) Sarcomere length shortening and (D) Active force normalised to maximum of Control.

Fig 2 shows the simulated electromechanical activities of the right atrial baseline model under control (states 1 & 2) and AFER conditions (states 3 & 4) for AP (Fig 2A), ${\left[Ca\right]}_{i}^{2+}$ (Fig 2B), SL (Fig 2C) and normalised active force (Fig 2D). In simulations, AFER abbreviated the atrial action potential duration. The measured APD_90_ (action potential duration at 90% repolarisation) values were 274 ms and 188 ms under control and AFER conditions respectively. This represented about 31% APD abbreviation by AFER, which was within the range of observed APD abbreviation in atrial cells of AF patients as compared to those of patients in sinus rhythm [10–12,14,24,87,88]. In simulations, AFER reduced the systolic ${\left[Ca\right]}_{i}^{2+}$ level by 25% and the diastolic ${\left[Ca\right]}_{i}^{2+}$ level by ~50% (Fig 2B), which was also within the range of observed reduction in systolic ${\left[Ca\right]}_{i}^{2+}$ transient amplitude in atrial cells of AF patients as compared to those of patients with normal sinus rhythm [14,88–91]. Fig 2B inset shows a comparison of the simulation APD_90_ and systolic ${\left[Ca\right]}_{i}^{2+}$ level reduction (grey bars) with relevant experimental data (symbols) from literature [10–12,14,87,88]. The reduced ${\left[Ca\right]}_{i}^{2+}$ transient amplitude led to a decreased sarcomere length shortening (Fig 2C) and consequently, to an 82% decrease in active force (Fig 2D). This reduction in active force is close to clinical data showing that in AF patients the average force of atrial contraction was reduced by about 75% [92]. The match between simulation data and clinical data at the cellular level for the reduced active force also provides validation for the use of the single cell model for the electro-mechanical coupling.

**Fig 3:**
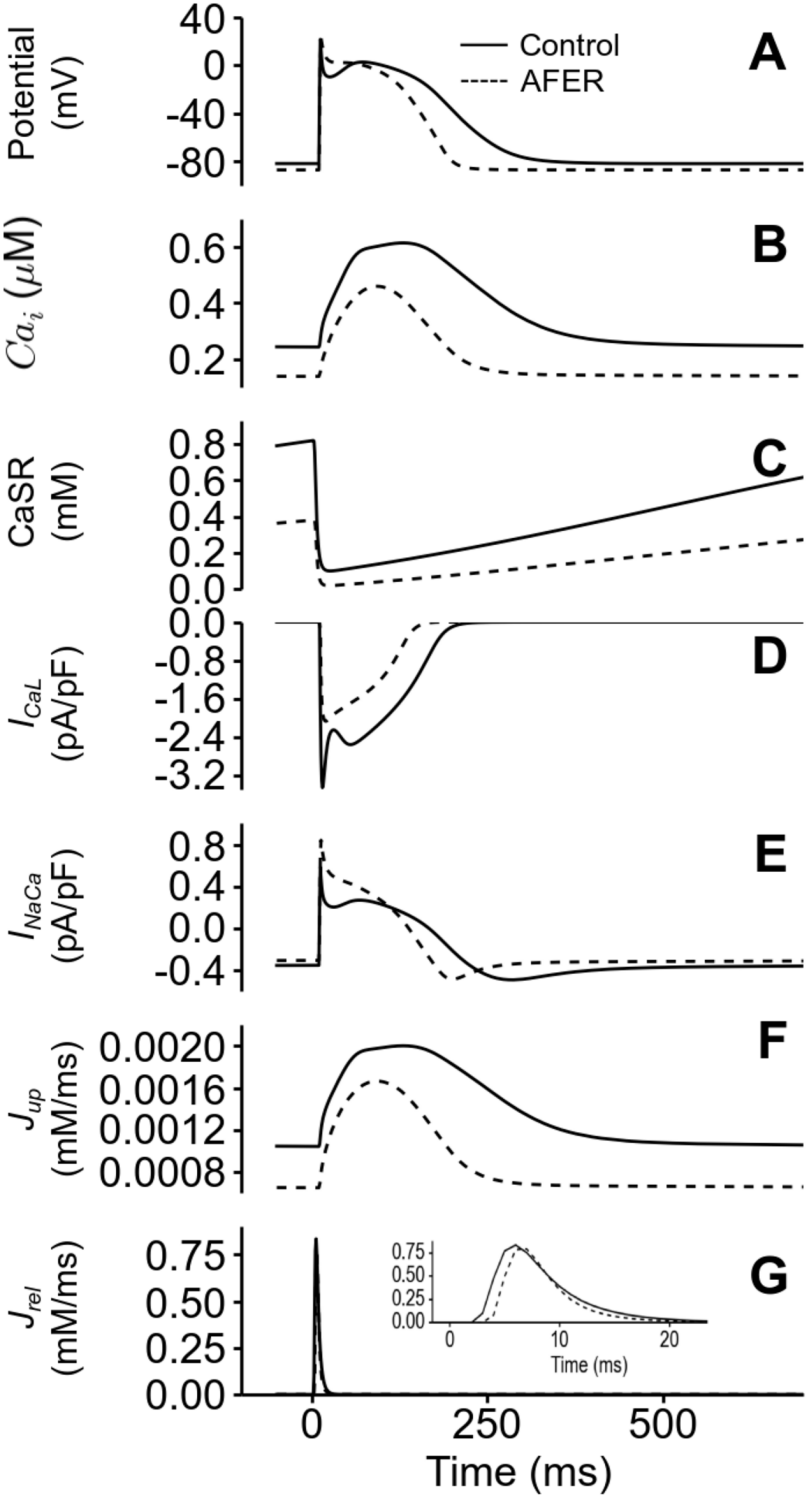
AFER effects on the Ca^2+^ signalling processes of the baseline, isolated RA single cell under control (dark) and AFER (dashed) conditions. (A) Action Potential. (B) Cytosolic Ca^2+^ concentration. (C) SR Ca^2+^ content. (D) L-type Ca^2+^ current (I_CaL_) density. (E) Na^+^/Ca^2+^ exchanger current density (I_NaCa_). (F) Flux of Ca^2+^ uptake into the SR (J_up_) and (G) Flux of Ca^2+^ release from the SR (J_rel_) Inset: Magnified view of J_rel_ in the first 20 ms.

The mechanisms for the reduced amplitude and diastolic level of ${\left[Ca\right]}_{i}^{2+}$ are attributable to the effects of AFER on the Ca^2+^ signalling processes as shown in Fig 3. The figure plotted the time courses under control (states 1 & 2) and AFER conditions (states 3 & 4) for AP (Fig 3A), ${\left[Ca\right]}_{i}^{2+}$ (Fig 3B), sarcoplasmic reticulum Ca^2+^ content (CaSR; Fig 3C), the L-type Ca^2+^ current density, (I_CaL_; Fig 3D), the Na^+^/Ca^2+^ exchanger current density (I_NaCa_; Fig 3E), the flux of Ca^2+^ uptake into the sarcoplasmic reticulum (J_up_; Fig 3F) and the flux of Ca^2+^ release from the sarcoplasmic reticulum (J_rel_; Fig 3G). It was shown that AFER reduced the CaSR content (by ~50%) (Fig 3C), which was accompanied by a reduced I_CaL_ (Fig 3D) that led to a reduced trigger for Ca^2+^ release from the sarcoplasmic reticulum, an increased Na^+^-Ca^2+^ exchange current, and a functionally reduced sarcoplasmic reticulum Ca^2+^ uptake and release though both maximal activities were increased by the AFER (see Table 1). All of these changes were collectively responsible for the AFER-induced reduction in the systolic and diastolic levels of ${\left[Ca\right]}_{i}^{2+}$.

**Fig 4:**
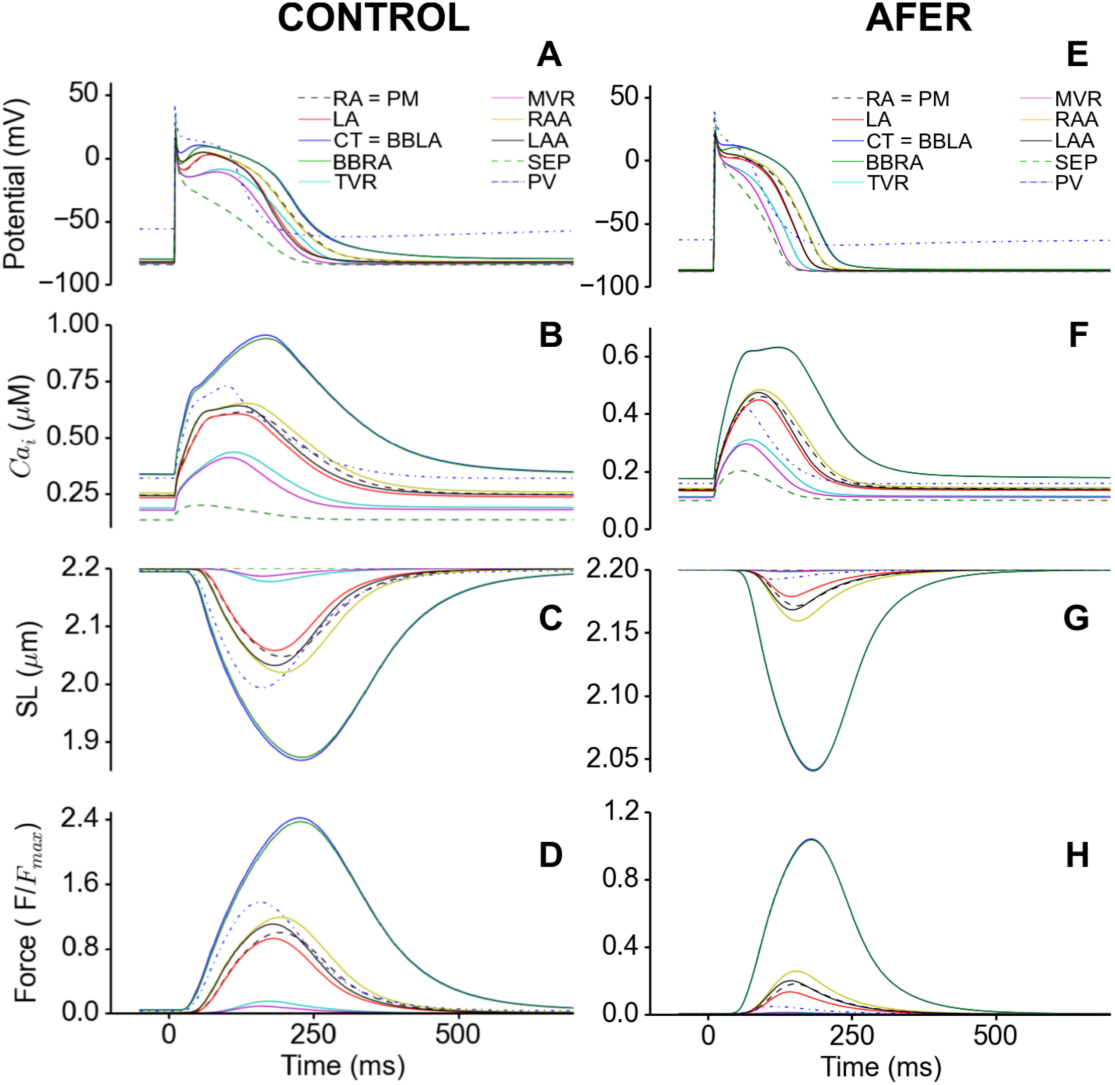
Electromechanical properties of the isolated regional atrial single cell types under control (A-D) and AFER (E-H) conditions. (A,E) Action potential. (B,F) Cytosolic Ca^2+^ concentration. (C,G) Sarcomere length shortening and (D,H) Active force normalised to maximum of Control (Note the different scales between control and AFER).

AFER also altered the electromechanical activities of other types of atrial cells as shown in Fig 4, which were created from the right atrial baseline model (Fig 2). In the figure, the electro-mechanical activities of different atrial cell types in the control condition (Fig 4A and 4D) are compared with those in the AFER (Fig 4E and 4H) conditions. The APs from the single cell family under both the control (Fig 4A) and the AFER conditions (Fig 4A) exhibited heterogeneous morphologies with significant differences in the diastolic and systolic ${\left[Ca\right]}_{i}^{2+}$ levels (Fig 4B and 4F), SL shortening (Fig 4C and 4G), active force (Fig 4D and 4H) and APD_90_, the latter ranging from 181 ms in the PV to 325 ms in the BBRA (Table 3). For all of the cell types, AFER abbreviated APD, reduced the diastolic and systolic level of [Ca^2+^]_i_, leading to reduced cell length shortening and the production of the active force. In simulations, AFER induced marked and heterogeneous reduction in the active force among variant cell types, ranging from a 57% reduction in the BBRA to 97% in the PV. There was negligible contraction in the SEP. Table 3 summarises the characteristics of the electromechanical activities for different cell models and the changes between the control and AFER conditions. Note that the range of simulated reduction in active force (57–97%) among variant types of atrial cells is also close to the clinical data of reduced force of atrial contraction observed in AF patients [92].

**Table 3:** Electromechanical properties of the family of single cell atrial models.

Cell Type	APD_90_ (Control) (ms)	APD_90_ (AF) (ms)	${\left[Ca\right]}_{i}^{2+}$ % Change (Control to AF)	Active Force % Change (Control to AF)
**RA/PM**	274	188	25%↓	82%↓
**LA**	234	167	26%↓	86%↓
**CT/BBLA**	322	213	34%↓	57%↓
**BBRA**	325	215	33%↓	56%↓
**TVR**	250	149	29%↓	93%↓
**MVR**	218	130	28%↓	92%↓
**RAA**	272	189	26%↓	78%↓
**LAA**	229	167	26%↓	82%↓
**PV**	181	145	43%↓	97%↓
**SEP**	189	125	26%↓	0%↓

RA: Right Atrium, LA: Left Atrium, PM: Pectinate Muscle, CT: Crista Terminalis, BB: Bachmann’s Bundle, TVR: Tricuspid Valve Ring, MVR: Mitral Valve Ring, RAA: Right Atrial Appendage, LAA: Left Atrial Appendage, PV: Pulmonary Vein, SEP: Septum

### 3D Electro-mechanical simulations in the 3D model

Atrial contraction is an integral action of a large population of myocytes that are electrically and mechanically coupled. Due to the complex geometry, heterogeneity and anisotropic properties of atrial tissue, it cannot be assumed that behaviour at the single cell level necessarily translates into similar activity at the organ level. In particular, reduction in cell shortening as observed in Fig 4 cannot automatically be interpreted as leading to reduction of the contraction volume of the atria, which is normally measured at the clinical setting: cells with differing electrophysiological and electromechanical properties are coupled, which will therefore influence timing of electrical propagation and therefore mechanical activity. In addition, passive stress arising from tissue contraction may also influence atrial contraction at the whole organ level. Thus, it is necessary to investigate the functional consequences of AFER at the 3D atrial organ level. Therefore, we performed further simulations using realistic human 3D anatomical atrial geometry with consideration of the intrinsic electrical heterogeneity in the atria.

**Fig 5:**
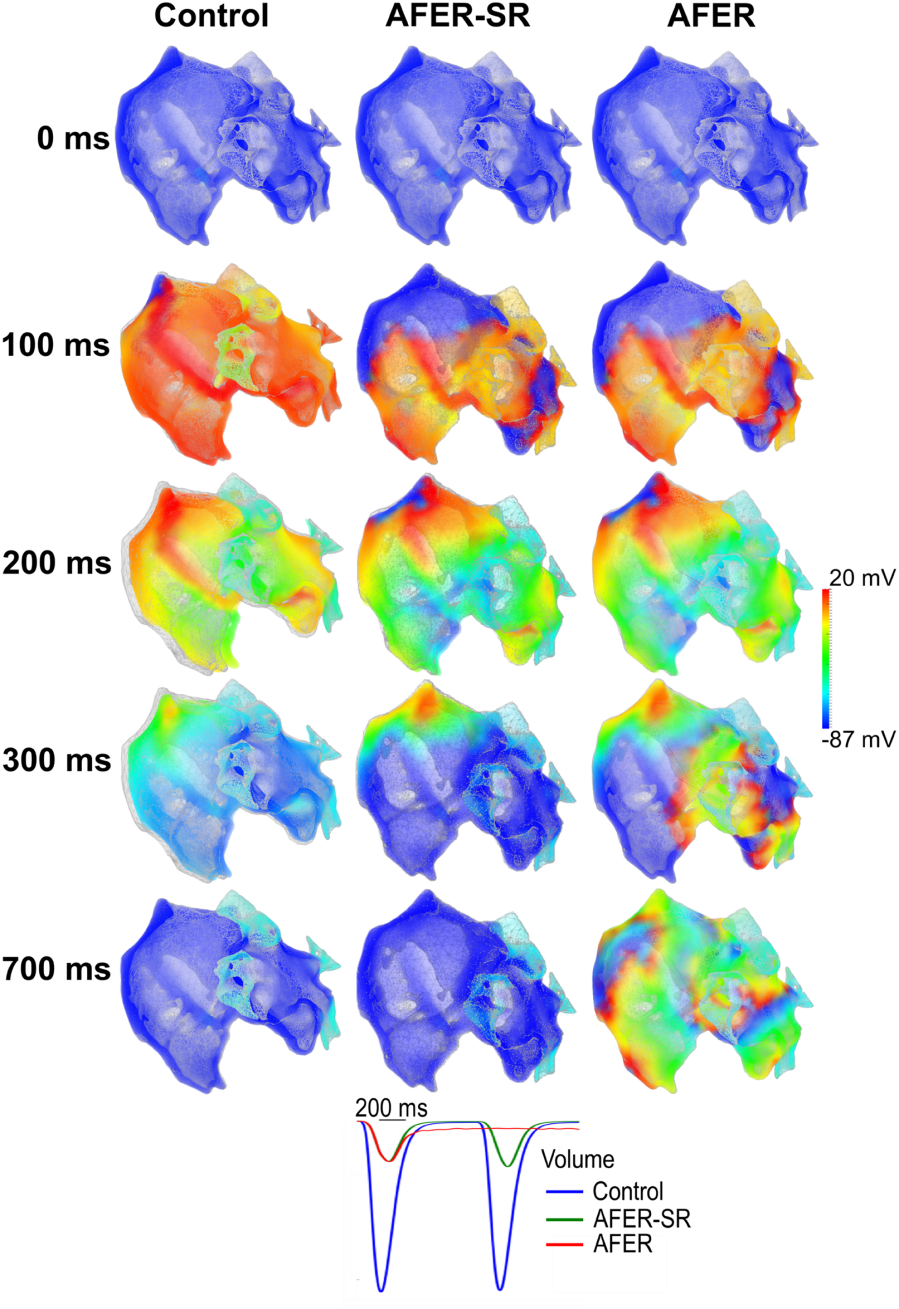
Snapshots of atrial electromechanical contraction superimposed on an atrial mesh (grey) under control and the tissue 2–3 months after cardioversion (Left; states 1 & 2), AFER-SR (Middle; AF-remodelled tissue under sinus rhythm; state 3) and AFER-AF (Right; AF-remodelled tissue with multiple re-entrant excitation waves; state 4) conditions. **0 ms:** Before electrical activation. **100 ms:** Almost complete activation under control (left) condition compared to slower activation in AFER-SR (middle) and AFER-AF (right) conditions. **200 ms:** Control (left) condition undergoing contraction, AFER-SR (middle) and AFER-AF (right) conditions with negligible contraction. **300 ms:** Control (left) and AFER-SR (middle) conditions undergoing repolarisation and re-entrant wavelets in the AFER-AF (right) condition. **700 ms:** Complete repolarisation and tissue relaxation in control (left) and AFER-SR (middle) conditions but multiple sustaining re-entrant wavelets in the AFER-AF (right) condition. **Bottom:** Time course of computed atrial volume during electrical excitation for control (blue), AFER-SR (green) and AFER-AF (red) conditions.

At the whole organ level, AFER impaired atrial mechanical contraction as shown in Fig 5, in which the sequence of electrical wave initiation and propagation, and the resultant contraction of the atria are shown for control (states 1 & 2; S1 Video), AFER tissue in the normal sinus rhythm (i.e, 1Hz) (AFER-SR) (middle panels for state 3; S2 Video) and AFER tissue with reentrant excitation wave condition (AFER-AF) (right panels for state 4; S3 Video). In all the cases, the deforming mesh was superimposed on an undeformed mesh (grey) in order to show the atrial contraction. At 0 ms, excitation was initiated from the SAN for both the control and AFER-SR conditions. At 100 ms, under the control condition, the whole atria had almost completely activated while in AFER, due to the slower electrical wave conduction, large parts of the right and left atria (blue-coloured) were yet to be activated. AFER caused a delayed atrial activation.

At 200 ms, there was significant atrial contraction under the control condition but comparatively negligible contraction under AFER-SR. There was a delayed onset of atrial contraction in the AFER condition.

At 300 ms, relaxation was underway under the control condition, but in the AFER-SR condition, relaxation was almost complete, suggesting a shortened contraction time course under AFER condition due to an abbreviated APD and the significant reduction in SR loading (Fig 3C) leading to an abbreviated time course of the intracellular Ca^2+^ transient.

Fig 5 also shows the time course of the atrial volume change during atrial excitation and contraction under both control and AFER-SR conditions. Volume change (i.e., atrial contraction) was reduced by 76.3% in the AFER-SR condition as compared to the control condition. Such a reduction in the atrial contraction volume was attributable to the reduction in active force at the cellular level (~80%) in all of the atrial cell types in the AFER condition (Table 3 and Fig 4). Note that at the 3D whole atria level, the simulation result of 76.3% reduction in atrial contraction corresponds with clinical data of 75% reduction of atrial contraction in AF patients [92], which provides further validation of the cellular and 3D organ models used for the electro-mechanical coupling of the human atria.

Further simulations were carried out to investigate the functional impact of AFER on atrial contraction while there were rapid reentrant excitation waves in the atria (AFER-AF; Fig 5, right panels). In this case, reentrant excitation waves were initiated by a premature stimulus applied to a localized region (IAS) at a 250 ms delay from the initiation of sinoatrial node excitation. Before the premature stimulus, the activation pattern and the mechanical contraction time course were the same as those in the AFER-SR condition. After the premature stimulus, sustained reentrant excitation wavelets developed, which were uncoordinated across the atria, leading to diminished mechanical contraction of the atria as illustrated by an almost flattened time course of the atrial volume.

**Fig 6:**
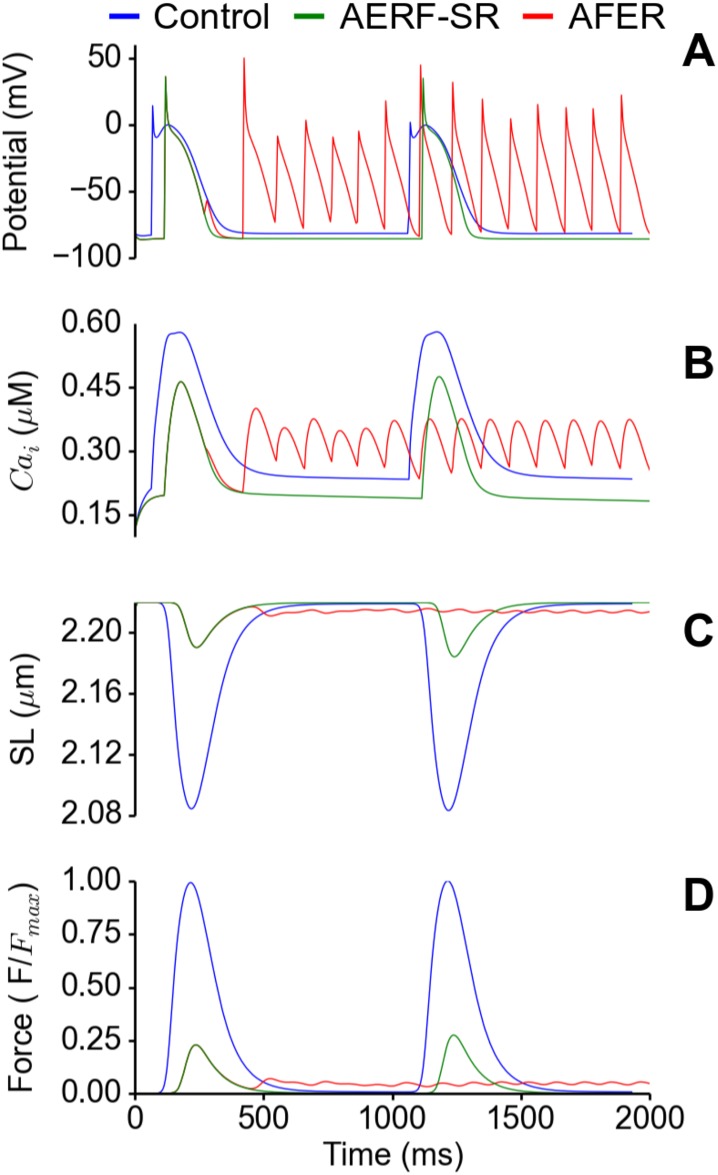
Electromechanical properties of a RA cell in the 3D atria under control (blue), AFER-SR (sinus rhythm, green) and AFER-AF (red) conditions. (A) Action potential. (B) Cytosolic Ca^2+^ concentration. (C) Sarcomere length shortening and (D) Active force normalised to maximum of Control.

Fig 6 shows the time course of AP (Fig 6A), [Ca^2+^]_i_ (Fig 6B), SL (Fig 6C) and active force (Fig 6D) recorded from a localized site of the atria under control, AFER-SR and AFER-AF conditions. It was shown that as compared to the control condition, AFER impaired atrial mechanical activity by reducing the diastolic and systolic levels of [Ca^2+^]_i_, leading to reduced cell length shortening and less active force production. For the AFER tissue with rapid reentrant excitation activity, though the diastolic [Ca^2+^]_i_ level was elevated because of Ca^2+^ accumulation as compared to the sinoatrial node rhythm, the systolic [Ca^2+^]_i_ level was markedly reduced, which is responsible for the reduced cell length shortening and the product of the active force.

## Discussion

In this study, we have developed a new biophysically detailed, 3D anatomical model of the electromechanical activity of the human atria. This incorporates accurate anatomical structures (Fig 1A) including fibre orientations (Fig 1B), which were validated against patient-specific volumetric models derived from CT, MRI and photographic data [43,44]. The 3D geometry itself is based on the Visible Female dataset [42]. The 3D human atrial electromechanical model also incorporates the intrinsic electrophysiological heterogeneity of the human atria through the integration of a family of heterogeneous, cellular models for the electrical APs of human atrial myocytes (Fig 3A and 3D). These cellular models were also modified to reflect ionic channel changes during chronic AF-remodelling (Fig 3E and 3H).

### Effects of AFER on atrial electro-mechanics

To investigate the effects of AFER on atrial electro-mechanics, simulations have been performed for 3 conditions mimicking 4 states. At the cellular level, AFER (state 3 & 4) abbreviated atrial action potential duration and reduced Ca^2+^ loading in the sarcolemma reticulum, which resulted in reduced diastolic and systolic [Ca^2+^]_i_ levels, leading to reduced cell shortening and active force production.

Impaired electro-mechanical activity at the cellular level was reflected by impaired mechanical contraction of the atria at the whole organ level for the AFER-SR condition (state 3). In simulations, AFER-SR resulted in a reduction in the emptying fraction of the atria as demonstrated by results shown in Fig 5. In the AFER-SR, the conduction of atrial excitation was slowed down, leading to delayed onset but earlier completion of atrial contraction as compared to the control tissue. The overall contraction volume was markedly reduced (Fig 5). Such impaired mechanical contraction is attributable in our model to the reduced level of diastolic and systolic [Ca^2+^]_i_ levels, resulting in reduced cell length shortening and production of the active force (Fig 6).

In our simulations of the AFER-AF tissue (state 4), multiple rapid reentrant excitation waves were initiated and sustained. Due to uncoordinated regional excitation waves, the mechanical contraction of the atria is significantly diminished even though the diastolic [Ca^2+^]_i_ level was elevated arising from Ca^2+^ accumulation (Figs 5 and 6).

### Relevance to Previous Studies

This study is the first to develop a 3D electromechanical model of the human atria with heterogeneous, electromechanically coupled, regional single cell models. It is also the first to investigate the cellular mechanisms responsible for weak atrial contraction in AFER tissue. Fritz *et al*. [93] developed a 3D electromechanical model of the human atria, but that study used a geometric model and appears to not consider atrial cellular heterogeneity, as it used just the right atrial CRN single cell model. Also it did not investigate AF [93]. Chapelle *et al*. [94] and Collin *et al*. [95] developed a surface-based electrophysiology model of the atria, treating the atria as a shell structure. Their model relied on detailed asymptotic analysis, reduced the computational cost of simulating a 3D atria and they presented strong convergence results of the numerical solution. However, they did not consider atrial deformation. Coudiere *et al*. [96] *et al*. also developed a two-layered model of atrial electrophysiology with two coupled, superimposed layers with the introduction of 3D heterogeneity. They used the model to explore numerical convergence for vanishing thicknesses. This model also reduced computational cost relative to a 3D model.

It is important to note that while multiscale simulations of atrial electromechanics are rare, there are several studies involving the ventricles and the whole heart such as the Multi-scale, Multi-physics Heart Simulator, “UT Heart” [97], the parallel multiphysics code for Computational Biomechanics, ALYA Red [98,99], the interactive framework for rehearsal of and training in cardiac catheter ablation from INRIA [100] and several others [57,68,101–103].

### Clinical Implications

Altered electro-mechanical function has important clinical implications. The failure of a return of atrial contraction following successful cardioversion may result in reduced exercise capacity and to an increased risk of thrombo-embolism and stroke. In addition, atrial stunning is associated with a greater risk of recurrence of atrial fibrillation [104], perhaps related to lower atrial pressures and less atrial stretch.

### Limitations

In addition to acknowledged limitations of both the CRN electrophysiology model [31] on which the atrial single cells were based and of the Rice *et al*. [36] mechanics model, all the other regional cell models used here are inherited from Colman *et al*. [41] atrial models, the limitations of which have been discussed in our previous study.[41]

The CRN electrophysiology model [31] is based on human experimental data whilst the Rice *et al*. myofilament model [36] is based on data from rat and rabbit. Ideally, all model parameters should be derived from human data, but as is often the case, there are a very limited amount of human experimental data to use to constrain the myofilament model and indeed the electromechanical model. This is a limitation but a necessary model compromise. This approach is consistent with that adopted in prior studies from our own group and others [41,105–107]. Therefore, qualitatively, at least, the model enables the elucidation of the contractile response under pathological conditions such as AF. Additionally, all temperature-dependent parameters were adapted to 37°C.

In the tissue model, intrinsic electrical heterogeneity and anisotropic intercellular coupling were introduced for several atrial regions phenomenologically [26,41,42,108] due to the lack of detailed experimental data from human atria. Atrial pressure is considered to be constant in the LA and RA but realistically, it varies throughout the cardiac cycle and may increase following the onset of atrial fibrillation. The use of a computational fluid dynamics model enabling the consideration of blood flow would allow the use of a more realistic pressure profile. The model does not take into consideration contact between the atria and the ventricles or the effect of ventricular motion on atrial mechanics, which all likely play a significant role in determining atria function. We did not investigate the effect of stunning on the LA and RA individually. It is possible that it affects both differently [8]. Therefore, this should be a subject of future study. Note that in simulations we have assumed that atrial tissue would take about 6 weeks (a time required for restoring atrial mechanical contraction after cardioversion) to complete reverse electrical remodeling, and simulated the tissue at that time using the same condition as healthy tissue. Though in animal models it has been demonstrated that reverse cardiac electrical remodeling process may take approximately the same time period [109], in human, due to multiple factors (structural, pathological and physiological) associated with atrial fibrillation, such an assumption may be too idealised. Whilst it is important that these potential limitations are stated, they do not fundamentally alter the principal conclusions of this study.

## Conclusion

We have developed an anatomically accurate, 3D biophysically detailed, electro-mechanical model of the human atria, which incorporates a suite of electro-mechanical single cell models comprising the different regions of the human atria. The model also incorporates fibre orientations validated against patient-specific data. Using the model, we have investigated the functional consequences of AFER for atrial contraction. We have shown that chronic AF-induced remodeling of ionic channel and intracellular Ca handling may be responsible for weak atrial contraction, a phenomenon as seen in atrial stunning after successful cardioversion.

## Supporting Information

S1 Video Atrial electrical wave propagation and mechanical contraction in the normal tissue (state 1) and the tissue 2–3 months after cardioversion (state 2). (AVI)

S2 Video Atrial electrical wave propagation and mechanical contraction under the atrial fibrillation-induced electrical remodelling condition under sinus rhythm (AFER-SR). (AVI)

S3 Video Atrial electrical wave propagation and mechanical contraction under the atrial fibrillation-induced electrical remodelling condition with atrial fibrillation re-entrant excitation waves (AFER-AF). (AVI)
